# Mid-term outcomes for Endoscopic versus Open Vein Harvest: a case control study

**DOI:** 10.1186/1749-8090-5-44

**Published:** 2010-05-28

**Authors:** Bilal H Kirmani, James B Barnard, Faisal Mourad, Nadene Blakeman, Karen Chetcuti, Joseph Zacharias

**Affiliations:** 1 Department of Cardiothoracic Surgery, Lancashire Cardiac Centre, Blackpool Victoria Hospital, Whinney Heys Rd, Blackpool, Lancashire, UK

## Abstract

**Background:**

Saphenous vein remains the most common conduit for coronary artery bypass grafting with increasing uptake of minimally invasive harvesting techniques. While Endoscopic Vein Harvest (EVH) has been demonstrated to improve early morbidity compared to Open Vein Harvest (OVH), recent literature suggests that this may be at the expense of graft patency at one year and survival at three years.

**Methods:**

We undertook a retrospective single-centre, single-surgeon, case-control study of EVH (n = 89) and OVH (n = 182). The primary endpoint was death with secondary endpoints including acute coronary syndrome, revascularisation or other major adverse cardiac events. Freedom from angina, wound complications and self-rated health status were also assessed. Where repeat angiography had been performed, this was reviewed.

**Results:**

Both groups were well matched demographically and for peri-operative characteristics. All cause mortality was 2/89 (2%) and 11/182 (6%) in the EVH and OVH groups respectively. This was shown by Cox Log-Rank analysis to be non-significant (p = 0.65), even if adjusting for inpatient mortality (p = 0.74). There was no difference in the rates of freedom from angina (p = 1.00), re-admission (p = 0.78) or need for further anti-anginals (p = 1.00). There was a significant reduction in the incidence of leg wound infections and complications in the endoscopic group (EVH: 7%; OVH: 28%; p = 0.0008) and the skew of high patient self-rated health scores in the EVH group (61% compared to 52% in the open group) approached statistical significance (p = 0.06).

**Conclusions:**

While aware of the limitations of this small retrospective study, we are heartened by the preliminary results and consider our data to be justification for continuing to provide patients the opportunity to have minimally invasive conduit harvest in our centre. More robust evidence is still required to elucidate the implications of endoscopic techniques on conduit patency and patient outcome, but until the results of a large, prospective and randomised trial are available, we believe we can confidently offer our patients the option and benefits of EVH.

## Background

Coronary Artery Bypass Grafting (CABG) remains the most common procedure in cardiothoracic surgery in the United Kingdom [1] and saphenous vein is still the most common conduit [2]. Traditional methods of vein harvest, in which a wound is opened along the length of the long saphenous vein, often contribute significantly to patient morbidity [3,4]. The advent of endoscopic vein harvest (EVH) has allowed surgeons to minimise this and many studies have demonstrated significantly reduced pain, infection rates and hospital stays [5]. While saphenous vein harvested endoscopically has been shown to have histologically similar appearances compared to vein harvested by the open method [6], preliminary studies looking at endothelial changes at the cellular level have given a mixed opinion [7,8]. Early studies showed statistically non-significant differences in graft patency at 6 months [9], and similar rates of event-free survival at 5 years [10]. The technique has not, however, been put through a rigorous prospective randomised trial to demonstrate its efficacy on long-term graft patency or patient outcomes. This reflects the ethical and logistic dilemmas of repeat angiography for large cohorts of asymptomatic patients. Also absent from the literature is a large multicentre trial focussing on patient reported outcomes and health related quality of life between the two groups.

One recently published study suggests that endoscopically harvested vein may, in fact, be associated with higher rates of vein-graft failure at one year and higher rates of death, myocardial infarction and need for revascularisation at three years [11]. One year graft patency rates in the open vein harvest arm of this study were equivalent to previous 20% graft failure rates demonstrated elsewhere [12], but significantly higher in the endoscopic group. In this subgroup analysis from a multicentre trial, however, the experience of the EVH operator was variable, with many centres presumably in the infancy of their endoscopic projects. The other weakness was that the technique and equipment used were not standardised and this may have impacted on the results of the study.

Our aim was to examine local outcomes with EVH to justify continued use of the technique in our centre and to collate robust long-term follow-up data.

## Methods

We undertook a retrospective case-control study of all consecutive first-time isolated CABG using at least one vein graft from a single surgeon in our centre. A study group undergoing endoscopic vein harvest (EVH) and a control group having open vein harvest (OVH) were considered. Inclusion criteria were bypass grafting of at least two vessels by the consultant surgeon (JZ). Exclusion criteria were: previous cardiac surgery; concomitant valve or aortic surgery; use of radial arterial conduit; use of both open and endoscopically harvested conduits; and routine use of aprotinin.

Assuming the cited differences in graft patency [11] manifesting as clinical symptoms, a power calculation was performed, which calculated a sample size of 326 for both groups with a 95% confidence interval and a statistical power of 75%.

Endoscopic vein harvest was performed with VASOVIEW 6 or VASOVIEW 7 Endoscopic Vessel Harvesting Systems (Maquet Inc, Wayne, USA), using a carbon dioxide insufflation technique. 2,500 units of heparin were administered prior to application of CO_2_. Diathermy was employed to divide side branches in situ with titanium clips applied prior to grafting. In the standard open technique, side branches were tied and clipped. Intermittent, cold blood, antegrade cardioplegia was the predominant method of myocardial protection.

The primary outcome measure was mortality, which was determined by consulting the local civil registry for deaths. Secondary outcome measures included any other major adverse coronary event (MACE) including acute coronary syndrome, or need for revascularisation. Freedom from angina was also used a secondary outcome measure, for which patients were reviewed initially by telephone survey to assess symptoms, readmissions and use of new anti-anginals. Clinical history was used to establish angina and dyspnoea grades on the Canadian Cardiovascular Society (CCS) and New York Heart Association (NYHA) functional classifications. The patient was also asked to score pain in the leg and sternal wounds on a ten-point scale (0-none, 10-high) and their current general health on a five-point scale of self-rated health status (poor, fair, good, very good or excellent). They were also asked to compare their health at the time of questioning with the pre-operative status on a five-point scale (much worse, worse, the same, better, or much better). Where patients cited clinical events or had required further investigation or treatment, case-notes were reviewed and, where relevant, angiographic data examined.

Numerical variables were compared by means of Student's t-test for normally distributed data and Mann-Whitney for non-parametric data. Categorical data was compared by means of chi-squared or Fishers Exact tests. Statistical analysis of data was performed using Prism 5 for Mac (GraphPad Inc, California, USA). Patients in whom endoscopic vein harvest was intended but who required conversion to an open procedure were included in the open vein harvest group. Most conversions were early in the experience and often due to difficulty in finding the vein in the thigh. The quality of the vein would not therefore have been affected by the conversion.

## Results

### Demographics

**Figure 1:**
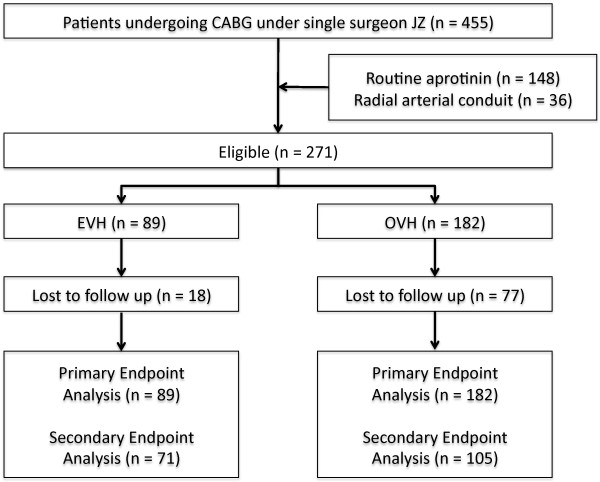
**Study profile**. CABG = coronary artery bypass grafting. EVH = endoscopic vein harvest. OVH = open vein harvest.

From the inclusion criteria, 455 eligible patients were identified. 148 were excluded as they had been operated on during a period of routine aprotinin use at the institution. A further 36 were excluded because of use of additional arterial conduits. Of the remaining 271 eligible patients, 89 had undergone endoscopic vein harvesting and 182 had undergone open vein harvesting (Figure 1). The median length of follow-up in the open vein harvest (OVH) group was 37 ± 6 months; and in the endoscopic vein harvest (EVH) group was 17 ± 7 months.

The two groups were demographically well matched although there was a significantly higher proportion of hypercholesterolaemia in the open vein harvesting group. In the endoscopic vein harvest group, there was a higher proportion of left main stem disease and proportionally fewer "good" left ventricular ejection fractions (Table 1). Operatively, the patients had similar bypass and cross-clamp times, although there were a smaller percentage of elective procedures in the open vein harvest group.

**Table 1:** Baseline characteristics of study groups

Characteristic	Total (n = 271)	Open Harvesting (n = 182)	Endoscopic Harvesting (n = 89)	p value
Age - yrs	66.1 ± 9.6	67.5 ± 9.7	66.0 ± 9.6	0.93
Male - no. (%)	223 (82)	145 (80)	78 (88)	0.13
Body Mass Index	28 ± 4.5	28 ± 4.3	29 ± 4.9	0.12
Hypertension - no. (%)	207 (76)	139 (76)	68 (85)	0.56
Diabetes - no. (%)				0.31
No diabetes	208 (77)	144 (79)	64 (72)	
Diet Controlled	9 (3)	4 (2)	5 (6)	
Tablet Controlled	41 (15)	27 (15)	14 (16)	
Insulin Dependent	13 (5)	7 (4)	6 (7)	
Hypercholesterolaemia - no. (%)	235 (87)	168 (92)	67 (75)	<0.001
Previous MI - no./total (%)				0.26
Last MI <30 days ago	27/140 (19)	22/97 (23)	5/43 (12)	
Last MI 31 - 90 days ago	18/140 (13)	13/97 (13)	5/43 (12)	
Last MI >90 days ago	95/140 (68)	62/97 (64)	33/43 (77)	
Prior PCI - no. (%)				0.64
None	248 (92)	166 (91)	83 (93)	
PCI >24 hrs prior to CABG	22 (8)	16 (9)	6 (7)	
PCI <24 hrs prior to CABG	0 (0)	0 (0)	0 (0)	
Previous stroke - no. (%)	23 (8)	16 (9)	7 (8)	1.00
Renal Failure - no. (%)	2 (1)	0 (0)	2 (2)	0.11
Peripheral Vascular Disease - no. (%)	40 (15)	26 (14)	14 (16)	0.86
Pulmonary Disease - no. (%)				0.46
None	240 (89)	159 (87)	81 (91)	
Asthma	16 (6)	13 (7)	3 (3)	
COPD	15 (5)	10 (4)	5 (6)	
Smoking - no. (%)				0.75
Never	91 (34)	60 (33)	31 (35)	
Ex-smoker	145 (53)	100 (55)	45 (50)	
Currently smoking	35 (13)	22 (12)	13 (15)	
CCS grade - no. (%)				0.55
I	53 (20)	41 (23)	12 (13)	
II	102 (38)	68 (37)	34 (38)	
III	98 (36)	66 (36)	32 (36)	
IV	27 (10)	20 (11)	7 (8)	
NYHA grade - no. (%)				0.64
I	96 (35)	72 (40)	24 (27)	
II	117 (43)	79 (43)	38 (43)	
III	82 (30)	57 (31)	25 (28)	
IV	11 (4)	7 (4)	4 (4)	
LMS disease >50% - no. (%)	68 (25)	37 (20)	31 (35)	0.0165
Left Ventricular Function - no. (%)				0.03
Poor <30%	10 (4)	7 (4)	3 (3)	
Fair 31-49%	44 (16)	22 (12)	22 (25)	
Good >50%	217 (80)	153 (84)	64 (72)	
Number of distal grafts	3.4 ± 0.8	3.5 ± 0.8	3.3 ± 0.9	0.24
Parsonett Score	8.3 ± 6.6	8.6 ± 6.9	8.2 ± 6.4	0.67
EuroSCORE	3.4 ± 2.3	3.4 ± 2.5	3.2 ± 1.9	0.50
Bypass time - min	83.3 ± 23.9	81.9 ± 22.5	86.3 ± 26.5	0.15
Cross-clamp time - min	64.9 ± 18.8	64.0 ± 17.7	66.8 ± 20.9	0.24
Operative Priority - no. (%)				0.0014
Elective	232 (86)	146 (80)	86 (97)	
Urgent	36 (13)	33 (18)	3 (3)	
Emergency	3 (1)	3 (2)	0 (0)	

### Outcomes

**Figure 2:**
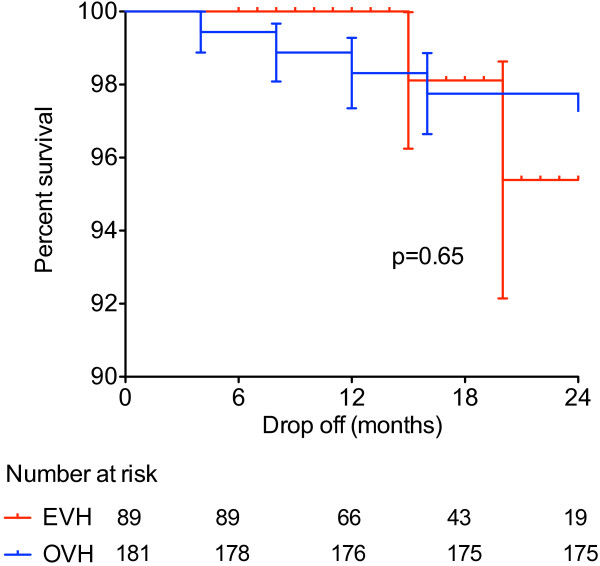
**Kaplan Meier Curve showing all-cause out of hospital mortality**.

Data for the primary outcome measure of death was taken from the civil registry and therefore follow-up was complete in all patients. All cause mortality in the endoscopic vein harvest group was 2/89 (2%) and in the open vein harvest was 11/182 (6%). Log rank analysis from a Kaplan-Meier survival estimation showed that there was no statistically significant difference (p = 0.65) between endoscopic and open vein harvest. Adjusting for early mortality within thirty days (Figure 2) which was 0/89 and 4/182 in the EVH and OVH groups, respectively, did not affect the statistical significance (p = 0.74). Cause of death for both groups was predominantly non-cardiac although four of the deaths in the open vein harvest group were not accounted for by post-mortem (Table 2).

**Table 2:** All-cause mortality

OVH group (n = 10) Cause of death (time since op/months)	EVH group (n = 2) Cause of death (time since op/months)
1. Multi-organ failure (inpatient)	1. Pancreatic carcinoma (15)
2. Aspiration pneumonia, ileus (inpatient)	2. Hepatocellular carcinoma (20)
3. Indeterminate (1)	
4. Haemorrhage from aortic cannulation site (1)	
5. Cerebrovascular accident (4)	
6. Cerebral atrophy (16)	
7. Indeterminate (8)	
8. Indeterminate (12)	
9. Prostate Carcinoma (25)	
10. Multiple Myeloma (35)	
11. Indeterminate (39)	

Clinical follow up was possible in 105 patients (58%) in the open vein harvest group and 71 patients (80%) in the endoscopic group. The remainder were lost to follow-up at the point of telephone interview.

In both study groups, there was a statistically significant reduction in angina and dyspnoea grades after CABG as compared to pre-op (Table 3). Patients in the endoscopic vein harvest group reported significantly fewer problems with leg wounds, with less antibiotic usage and district nurse involvement for delayed wound healing (Table 4). Pain scores for both the leg and the sternal wounds were not remarkably different between the two groups although the difference was statistically significant (p < 0.0001). There was no difference between the two groups in requirements for new anti-anginals (p = 1.00) or in the rates of re-admission with cardiac problems (7% in the EVH group and 9% in the OVH group) (p = 0.78).

**Table 3:** Angina and dyspnoea grading pre- and post-operatively

Characteristic	Open Harvesting (n = 105)	Endoscopic Harvesting (n = 71)	
CCS grade			
Pre-op	2.1 ± 1.1	2.2 ± 1.0	
Post-op	0.2 ± 0.6	0.2 ± 0.7	P = 0.95
	P < 0.0001	P < 0.0001	
			
NYHA grade			
Pre-op	1.9 ± 0.8	2.1 ± 0.8	
Post-op	0.5 ± 0.9	0.9 ± 0.9	P = 0.0013
	P < 0.0001	P < 0.0001	

**Table 4:** Post-operative complications and recurrences of symptoms

Characteristic	Open Harvesting (n = 105)	Endoscopic Harvesting (n = 71)	P value
Leg wound infection - no. (%)	29 (28)	5 (7)	0.0008
Leg Wound pain	1.0 ± 2.3	1.3 ± 1.4	<0.0001
Sternal Wound pain	0.8 ± 0.9	1.6 ± 1.3	<0.0001
Need for new anti-anginals - no. (%)	13 (12)	9 (13)	1.00
Further cardiac admissions - no. (%)	9 (9)	5 (7)	0.78

The average (mode and median) response in the self-rated health-status was "very good" with 52% of patients in the OVH group and 61% in the EVH describing their general health as either very good or excellent. The differences in these distributions seemed to approach statistical significance (p = 0.06). Similarly, the average response for the comparative health-status was "much better" with 81% in the OVH and 90% in the EVH groups stating that they were "better" or "much better" symptomatically as compared to before CABG.

Two patients in the EVH group and three in the OVH group also reported having returned for repeat angiography. In the EVH group, one patient had both of their saphenous grafts patent; the other patient had a single vein graft out of four occluded and the remainder patent. Of the three patients in the OVH group, one patient had all-patent grafts; one patient had an involuted LIMA but patent saphenous vein grafts; and one had two occluded saphenous vein grafts to the right coronary artery and the first obtuse marginal (Table 5).

**Table 5:** Angiographic findings in symptomatic patients

OVH group (n = 3)		EVH group (n = 2)	
**Patient One**		**Patient One**	
LIMA - LAD	Patent	LIMA - LAD	Patent
SVG - OM1	Patent	SVG - OM2	Patent
SVG - RCA	Patent	SVG - RCA	Patent
			

**Patient Two**		**Patient Two**	
LIMA - Diag (sequential)	} LIMA involuted	LIMA - LAD	Patent
LIMA - LAD	} and stented	SVG - Diag	Patent
RIMA - RCA	Patent	SVG - OM1	Patent
SVG - OM1	Patent	SVG - OM2	Occluded
		SVG - RCA	Patent
			

**Patient Three**			
LIMA - LAD	Patent		
SVG - OM1	Occluded		
SVG - OM2	Patent		
SVG - RCA	Occluded		

LIMA - Left Internal Mammary Artery, LAD - Left Anterior Descending Coronary Artery, SVG - saphenous vein graft, OMx - x^th ^Obtuse Marginal, RCA - Right Coronary Artery, Diag - Diagonal

## Discussion

Our unit has been performing endoscopic vein harvest since 2007 in line with the current trend for minimally invasive surgery. With the publication of the subgroup analysis of the PREVENT IV Trial by Lopes et al, it was felt necessary to scrutinise our local outcomes and mortality in order to determine if we were doing our patients a disservice. A retrospective analysis of the cohort that had already undergone EVH was deemed to be the most appropriate way of reviewing our results. We opted for a case-control study from a single surgeon in order to minimise the number of confounding factors introduced by different surgical techniques or management. While the case and control cohorts were chronologically separated, any benefits conferred by the contemporary nature of endoscopic vein harvest were likely to be small as the time period encompassed less than ten years [12]. In addition, we aimed to minimise any significant changes to practice that occurred during this time. For this reason, we excluded 148 patients in whom aprotinin was used as a routine protocol who would otherwise have been included in the open vein harvest group. Although there was a significantly higher proportion of non-elective patients in the open vein harvest group, pre-operative risk stratification using EuroSCORE was similar.

It was expected with a single-centre, single-surgeon experience of newly adopted endoscopic vein harvest that our sample size would fall short of statistical power, and we acknowledge the need for a larger study population and are in the process of contributing this data to a larger registry. In addition, the loss of patients to follow-up may have skewed results as those more willing or able to participate in follow-up could be assumed to have a better compliance with medical advice.

With these study limitations taken into consideration, our results demonstrate a reassuring clinical outcome in the medium term for endoscopic vein harvesting. We have included all our cases from the very first as even though there may be an acceptable learning curve in time taken [13] we feel - with the current improvements in technology, increasing adoption of minimally invasive procedures and support from the industry - that it is unacceptable to accept a reduction in conduit quality during the learning curve.

### Primary Outcomes

Our primary intention in undertaking this study was to investigate the possibility of endoscopic vein harvest adversely affecting survival and graft patency compared to open vein harvest. Lopes et al made a valid criticism of early studies, pointing out that many included patients in follow up for 4 to 6 weeks after surgery whereas the divergence in outcomes did not seem to manifest until one year. Our results demonstrate no difference in mortality, freedom from angina or major adverse cardiac events between the two groups at a median follow up of 17 months. Similar results have recently been described by Ouzounian, et al [14].

Freedom from angina is employed in this study as a surrogate marker of graft patency, although it is known that a significant proportion of asymptomatic patients may have graft occlusion [15] and that recurrence of symptoms is not necessarily an indication of graft failure [16,17]. It is not clear, however, what the clinical implications of asymptomatic graft failure are, as data from trials in which angiography is incorporated into the study protocol may demonstrate twice as much graft failure as that seen in angiography for symptoms [18]. The management of asymptomatic graft stenosis or occlusion remains contentious as graft PCI and re-do CABG carry higher risk burdens [19]. Conversely, progression of atherosclerosis in saphenous grafts is associated with increased risk for subsequent coronary events independent of symptoms [20].

Gaining ethical approval to conduct protocol-driven angiography for research purposes would be difficult in the United Kingdom. The merits of subjecting asymptomatic patients to a small but serious risk for the procedure are questionable, especially where management may not be altered. The use of non-invasive methods of angiography in asymptomatic patients has been demonstrated in the UK [15], but is probably not yet advanced enough to replace traditional angiography [21].

### Secondary Outcomes

Our study reiterates the significant improvement in wound healing, complications and satisfaction after endoscopic conduit harvest. While the differences in pain scores were shown to be statistically significant (P < 0.0001) in favour of the traditional open technique, these differences were likely to be beneath the sensitivity threshold of the pain scale. The ten-point pain scale probably requires a "minimal important change" of Δ2 in order to be considered substantially different [22]. The lack of any pain-related benefits in endoscopic versus open harvest may also reflect the disparity in follow-up of time since operation.

Self-rated health status is dependent on additional factors such as socio-economic status [23] and psychological well-being [24], but has been shown to correlate well with long-term survival after angioplasty [25]. In this population, unmatched for psychosocial confounding factors, the differences in our two study groups provides interesting additional data, but we are cautious about interpreting the implications any further than patient satisfaction.

## Conclusions

Since its inception less than two decades ago, endoscopic vein harvest has become both widely adopted and a common expectation from patients. The accepted wisdom of minimal access conduit harvest has been called into question lately due to the publication of a subgroup analysis from the PREVENT IV Trial. Our review, despite its potential flaws, was helpful for us to justify continuing with our programme of EVH. We hope this will also reassure other centres currently reviewing the practice while we await the funding to carry out a long overdue prospective randomised trial looking specifically into the long term effects of endoscopic vein harvesting.

## Competing interests

The authors declare that they have no competing interests.

## Authors' contributions

All authors were responsible for conceiving and developing the study protocol; JZ & NB performed all EVH cases; KC, FM, BHK & NB conducted telephone interviews and collated data; BHK & JBB reviewed data and performed statistical calculations; BHK, JBB & JZ wrote the final manuscript. All authors read and approved the final manuscript.
